# Gene–culture interaction and the evolution of the human sense of fairness

**DOI:** 10.1038/srep32483

**Published:** 2016-08-26

**Authors:** Tru-Gin Liu, Yao Lu

**Affiliations:** 1 National Sun Yat-sen University, Institute of Economics, Kaohsiung, 80424, Taiwan; 2 Wuhan University, Dong Fureng School of Economic and Social Development, Beijing, 100010, China

## Abstract

How Darwinian evolution would produce creatures with the proclivity of Darwinian generosity, most of them voluntarily giving up the immediate benefit for themselves or their genes, remains a puzzle. This study targets a problem, the origin of human sense of fairness, and uses fairness-related genes and the social manipulation of Darwinian generosity as the key variables underlying the human sense of fairness, inequity aversion, as well as their relationships within cooperation, and the anticipation foresight of the way relationships are affected by resource division, given the assumption of randomly matched partners. Here we suggest a model in which phenotype will gradually converge towards the perfect sense of fairness along with the prospect of cooperation. Later, the sense of fairness will decrease but it is never extinct. Where social manipulation of Darwinian generosity overshadows genetics, the sense of fairness could be acute to the degree of social manipulation. Above all, there still exists a threshold in the degree of social manipulation, beyond which altruism dominates selfishness in human cooperation. Finally, we propose three new directions toward more realistic scenarios stimulated by recent development of the synergy between statistical physics, network science and evolutionary game theory.

The sense of fairness remains unknown how it arose, especially Darwinian generosity, observed only in chimpanzees and humans^1^,^2^. It argues that the human response to unfairness evolved in order to support long-term cooperation^1^. Gene–culture coevolution is key for explaining important patterns within the sense of fairness^3^. Neuroeconomists, recently, have been particularly interested in assessing the way allocation decisions are made while interacting with others, pointing towards how the sense of fairness emerges and works^2^,^4^. Evidence shows that negative effects on altruistic cooperation are caused by economic incentives in the case when they come in the form of sanctions or are associated with selfish intentions^3^. Because the identification of fairness-related genes escapes measurement, while genetic group selection is unlikely to occur empirically^5^,^6^, simulation experiments are focusing potential evolutionary patterns of fairness-related genes. Here we show that there exists a critical value in the degree of social manipulation, beyond which altruism dominates the perfect sense of fairness, and *vice versa*. Second, the emergence of the perfect sense of fairness is not necessarily reliant on continued intra-generational cooperation^1^. The increasing prospect for cooperation across generations is sufficient enough for its emergence. In contrast with the intra-generation version wherein reputation is important, the sense of fairness evolves intergenerationally as well^7^. Third, the sense of fairness, the first degree inequity aversion (IA_1_), and the second degree inequity aversion (IA_2_) are not mutually independent even without continued cooperation^1^. Furthermore, most results of the ultimate game experiments^2^,^8^,^9^,^10^,^11^,^12^,^13^,^14^,^15^,^16^,^17^ are consistent with the fairness-norm hypothesis^18^. In the more general context, social manipulation is a device, overshadowing the genetics system to associate people together wherever applicable. From a broader perspective, this device has far-reaching implications for the evolution of human sense of fairness in the near future.

Is sense of fairness innate or something developed in social life? The answer relates to what fairness is defined as in terms of evolution. The argument that vampire bats have their own way of sharing blood with nonkin can be made in complete analogy with our way of sharing, *i.e.*, “fairness”^19^. Following this thought, how our current fairness norms operate is indivisible from how they evolve, since each sheds light on the other. Although, only a small portion of mammals have ever demonstrated a sense of fairness or unfairness among all, a few primates and almost all breeds of apes do have this sense with the levels of IA varying upon the group of mammals. In most cases, they belong to IA_1_, negative reactions to inequity to the detriment of the actor^8^. Human’s economic incentives mainly cause negative effects on altruistic cooperation, if they come in the form of sanctions and are associated with greedy or selfish intentions^3^. IA_2_, also known as Darwinian generosity, refers to negative reactions to inequity that benefits the actor or overcompensator^2^. It requires advanced cognition and emotional control, only observed in chimpanzees and humans thus far. IA_1_ is reported to be more pronounced than IA_2_^20^, while IA_1_ emerged earlier as well^21^. In neuroeconomics, there recently has been a surge of interest in assessing the way allocation decisions are made while interacting with others, especially how the sense of fairness emerges and works^4^. This is the foundation of the full-blown human sense of fairness. Although fairness is essential to humans, how it arose remains unknown, especially, IA_2_.

Scientists believe that the emergence of the sense of fairness is in close relation with cooperation, despite presenting an evolutionary puzzle for its negative impact on the short-term interests of at least some parties. Theoretical and experimental studies on various guises of cooperation and a wide range of animal species suggest four paths to cooperation: reciprocal altruism, kinship, group-selected cooperation, and byproduct mutualism. Each can be transformed directly from the cooperator’s dilemma game with slight changes in the payoffs. Cooperation in a particular case may follow a single path or contain at least two elements^22^. Hence, the human sense of fairness should associate four dimensions, each with at least one path to cooperation. In addition, many studies emphasize that culture and social learning are substantially important in its formation^23^,^24^.

It argues that the human response to unfairness evolves from the sake of supporting long-term cooperation, otherwise the latter would not have sustained without appropriate channels to ensure the sharing of payoffs^1^. One wonders whether such channels are more associated with social environmental evolution or was already embedded within our genes.

Scholars believe that experimental results drawing from the pure ultimatum game (UG), wherein haggling is impossible, people do not get to know each other, the total sum disappears if not split on the first attempt, and the game plays just once, are helpful to uncover the fundamental principles governing human’s decision-making mechanisms^2^. In most studies on UG experiments undertaken in typical Western-type civilisations, the majority of proposers offer 40% to 50% of the total sum, and about half of all responders reject offers below 20%^2^,^8^,^9^,^10^,^11^,^12^,^13^,^14^,^15^,^16^. While the former is far from what rational analysis would dictate for selfish players, the latter has often been attributed to negative feelings toward perceived inequity^4^. In marked contrast to general reports, the findings from 15 small-scale societies across 4 continents show sizable variations in the offer. For example, the mean offer within the Machiguenga tribe in the Amazon was only 26%, but many members of the Au tribe in Papua New Guinea offered more than 50% although the Au tended to reject offers either excessively generous or miserly. Despite these variations, most people worldwide reveal a high value on fair outcomes^25^.

Rejecting low offers is exorbitant, and whether behaviour changes along with all stake levels is an inescapable question. It reports that stakes do not matter while low offers up to 10% to 20% have rarely been observed^26^,^27^. In contrast, some evidence shows that *sufficiently high stakes* lead responder behaviour to converge toward full acceptance of low offers even without learning when stakes calibration have been treated beforehand^28^. Various elements have been added to UG to suit for numerous situations so as to explore the causes of the emotional behaviour inconsistency thus elicited. For instance, in the cases where the competition for being a proposer is intense, inequity can be justified, or in other words, people get to know each other, which then lowers the amount of routine offers thus getting accepted much easier^2^,^29^. Nonetheless, these outcomes are far from what rational analysis would dictate for Homo economicus if only offers do not converge toward zero. On the other hand, rejecting a dismal offer involves an immediate cost, which may be offset by gains in future encounters from reputation acquired through an internal device, self-esteem. A repeated UG shows that fairness can evolve intra-generationally if associated with reputation^7^.

One of the modified UG experiments reports that neither the apes nor the 3~5-year-old human children consistently refused offers, but behavioural protest did occur^1^. It suggests that decision making in a socioemotional context relates to cognitive or affective processes. Research findings demonstrate that the influence of emotion on decision making deepens upon age^30^. This makes UG particularly valuable for assessing age differences in financial decision making, since emotion has its value over the share in a repeated game. Experimental results reveal that older adults divided the money more generously than young adults whom rejected more unfair offers proposed by young adults compared to older adults. Moreover, both participating age groups reported to be displeased at unfair offers proposed by young adults compared to receiving the same offer from an older adult^31^. As well, prosocial sentiments may be evident in the decisions of older responders, due to the previous noted self-serving motivation while accepting an unfair offer and the increasing recognition in age concerning the social goals of fairness and generosity^32^. The findings from various variants of UG experiments suggest that real people are a complicated hybrid species of Homo economicus and Homo emoticus. The major challenge is to theorize how Darwinian evolution would produce creatures with the proclivity of Darwinian generosity.

One class of experiments that comes close to UG is the dictator game. In one set of the experiment, dictators gave 1/3 of the total sum to the paired anonymous student^17^. Similarly, in “gangster” experiments, the gangster students took away “only” 3/4 of the total sum^33^,^34^. These findings inspire us to look to the potential link between social and genetic inheritances. Among the three classes of experiments, both the dictator game and the gangster game did not require cooperation between two randomly paired individuals. In the sort of UG, however, this cooperation is necessary to materialize the total sum. Therefore, insights drawing from UG experiments are valuable as far as the evolution of the human response to unfairness is concerned.

The sense of fairness is by itself a reflection of cognition. Recently, neuroscientists have attempted to unravel neural genes in the areas of the brain implicated with cognition. Current studies can only verify a handful of neural genes, all of which relating to behaviours more at an elementary level, not mentioning more abstract or advanced cognition. If the sense of fairness is not innate, then it can only be acquired from social inheritances. In this case, fairness-related genes should be overlooked since social cultural environment is partly extrasomatic. However, if the sense of fairness is innate, one would wonder how fairness-related genes will evolve. Will they converge over infinite gene-culture coevolution? Because the identification of fairness-related genes escapes measurement, it is difficult to conduct empirical tests that distinguish among various arguments. Hence, simulation experiments are conducive to demonstrate the evolution of fairness-related genes in the context of various patterns of gene-culture interactions.

Our controlled simulation experiments are carried out in an imaginary isolated island with a constant environment carrying a capacity of *N* individuals of each generation. Assume that *N* is evenly split between the male and female gender, and the costs of help and reciprocal exchange are identical among kin *versus* nonkin, so that there are no differences between reciprocal altruism and kinship in terms of the path to cooperation. The life of each individual must go through the stages of birth, preying, reproduction, and death. When an ovum meets a sperm, two strands of genes will crossover normally or mutate with a low probability to determine the unique phenotype of the fertilized ovum, which can be considered as an observable behavioural mode. In the second stage, an individual preys either alone or cooperatively with others. In the third stage, individuals with higher degree of fitness will have more offspring than others thus forming a dominant group in the population in the long run.

During the hunting period, each individual goes out as predators to find prey for *M* times totally. For each time’s outing for preying, an individual can realise a certain constant payoff, *π*_0_ > 0, if hunting singly. To cooperate with someone is the other alternative. Assume that individuals seeking for cooperation are repeatedly randomly matched in pairs. Assume further that the probability an individual is matched with the same opponent more than once is negligible, and it is not necessary for an individual to establish reputation or to give up an immediate benefit to stabilize a long-term valuable cooperative relationship. Suppose that perfect cooperation in the absence of IA will bring with the total net reward of *π*_1_ to be divided among participants. Without loss of generality, cooperation among three or more individuals are excluded, assume that *π*_1_ > 2*π*_0_ holds true to allow for positive net gain from perfect cooperation.

All individuals are assumed to make less optimal decisions and are programmed with a mixed behavioural mode, which is not considered as a conscious choice. As a result, an individual sometimes cooperates with someone to hunt, instead of preying alone constantly. To operate on this idea, assume that there are *m* times, *m* ≤ *M*, an individual will look for cooperation opportunities while capable of hunting. The tendency how *m* evolves across generations, *t*, becomes critical in justifying the argument that the sense of fairness relates to cooperation.

For each generation, an individual *i* is assumed to be reproduced by random mating. The genotypes of *i*’s fairness-related genes are denoted by (B_*i*_, Γ_*i*_, Θ_*i*_), with each operator in parentheses representing the genotypes of the sense of fairness, the IA_1_-discount factor, and genetic IA_2_ respectively. Unless otherwise noted, each genotype is a binary string of length 4. The phenotypes of B_*i*_ and Γ_*i*_, denoted as *b*_*i*_ and *γ*_*i*_, are simply the products of three genetic operators: reproduction, crossovers, and mutations. In addition, the phenotype of Θ_*i*_, denoted as *θ*_*i*_, is the product of gene-culture interaction. Denote the parameter *α* ∈ [0, 1] as the degree of social manipulation. The bijection from a gene-culture profile to the profile of phenotypes is set as (*b*_*i*_, *γ*_*i*_, *θ*_*i*_) = (B_*i*_ × Φ, Γ_*i*_ × Φ, *λ α* + (1 − *λ*)Θ_*i*_ × Φ), where *b*_*i*_, *γ*_*i*_, *θ*_*i*_, *α* ∈ [0, 1], *λ* ∈ {0, 1}, and Φ = (2^0^, 2^1^, 2^2^, 2^3^)^T^/(2^4^ − 1). Since the human sense of fairness is the focal point, only *b*_*i*_ is termed as the phenotype hereafter, which is further classified into five categories: pure altruism (*b*_*i*_ = 0), strong sense of fairness (0 < *b*_*i*_ < 0.5), perfect sense of fairness (*b*_*i*_ = 0.5), weak sense of fairness (0.5 < *b*_*i*_ < 1), and pure egoism (*b*_*i*_ = 1).

The Methods section will detail the connections among the profile of (*b*_*i*_, *γ*_*i*_, *θ*_*i*_), the magnitude and the division of total payoffs, each individual’s lifetime payoffs, as well as the realised genotype of the next generation. The following summarizes only the elements with direct bearings on the experiments undertaken.

Let *β*_*i*_ = *b*_*i*_/(*b*_*i*_ + *b*_*j*_) and *β*_*j*_ = 1 − *β*_*i*_. Denote Π_*i*_ as individual *i*’s payoff in each random encounter with *j*, which equals [*β*_*i*_ − *θ*_*i*_(*β*_*i*_ − *β*_*j*_)/2] *γ*_*j*_*π*_1_ if *b*_*i*_ + *b*_*j*_ ≤ 1 and *b*_*i*_ > *b*_*j*_ both hold; [*β*_*i*_ + *θ*_*j*_(*β*_*j*_ − *β*_*i*_)/2] *γ*_*i*_*π*_1_ if *b*_*i*_ + *b*_*j*_ ≤ 1 and *b*_*i*_ < *b*_*j*_ both hold; 0.5 *π*_1_ if *b*_*i*_ = *b*_*j*_ = 0.5 holds; *π*_0_ if otherwise, for all *i* ≠ *j*, *i*, *j* = 1, ..., *N*. Thus, the lifetime payoffs of individual *i* of the *t*^th^ generation can then be calculated, denoted as 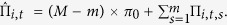 Assume that the ability to transmit one’s own genotypes is proportional to lifetime payoffs. Given *N*, the probability *a priori* of any individual *i* of the *t*^th^ generation transmitting her (his) own genotypes to the next generation can be determined, denoted as *P*_*i*,*t*+1_ = 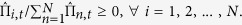 It helps determine the realised genotypes of the next generation through the operators of genetic reproduction, crossover, or mutation. Let *x* ∈ [0, 1] and *y* ∈ [0, 1] be the probabilities of crossover and mutation for each element of the inherited genes respectively.

Six controlled experiments of gene-culture algorithm simulations up to 10,000 generations are conducted, either with or without the social manipulation of IA_2_ to explore how the sense of fairness embedded in the phenotype evolves through genes alone or within gene-culture interactions. The first two experiments do not involve IA, aiming to examine whether the distribution of the phenotype is sensitive to changes in the prospect of cooperation upon convergence. The other four experiments are linked to IA_1_ or IA_2_ in various aspects, either with or without social manipulation of IA_2_, aiming to evaluate potential influences that different degrees of social manipulation of IA_2_ may bring about on the phenotype upon convergence. Note also that B_*i*_ is extended to a binary string of a length of 8 to allow for more variants within population in the experiments where IA is absent or genetic IA_2_ is overshadowed by social manipulations.

## Results

All experiments have the common set of parameters: *π*_0_ = 0.01, *π*_1_ = 1, *N* = 1,000, *M* = 100, *x* = 0.5, and *y* = 0.01. To capture stakes effect, *π*_1_ is set at the value 100 times of *π*_0_. As well, *m* = *t*/100 except in the second experiment where *m* = max {100 − *t*, 0}.

### Two experiments without IA

**Figure 1:**
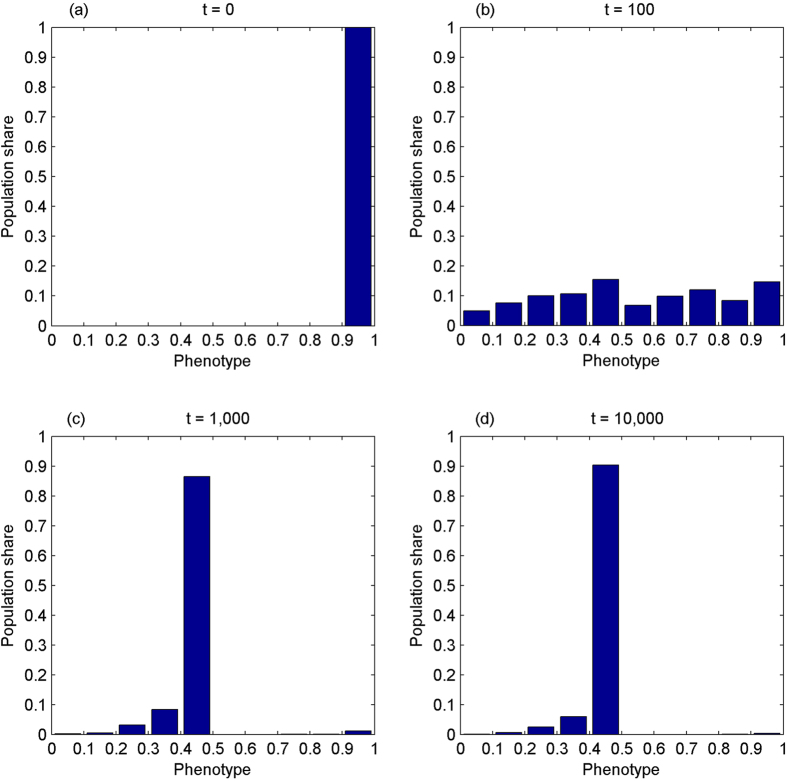
The phenotype evolves towards the perfect sense of fairness without inequity aversion while the amount of cooperation opportunities is increasing over time. It starts from the mass range of [0.9, 1], corresponding to nearly pure egoism, Panel (a). At t = 100, Panel (b), it spreads out more or less evenly. At t = 1,000, Panel (c), over 80% of population fall on the range of [0.4, 0.5]. At t = 10,000, Panel (d), it converges to the mass range of [0.4, 0.5], 90% of total population.

The first experiment simulates the evolution of the phenotype in the absence of IA while the number of cooperation opportunities is increasing over time. Assume that the phenotype starts from the mass range of [0.9, 1], corresponding to nearly pure egoism. As a result, the phenotype demonstrates a tendency to converge towards the perfect sense of fairness, see Fig. 1.

**Figure 2:**
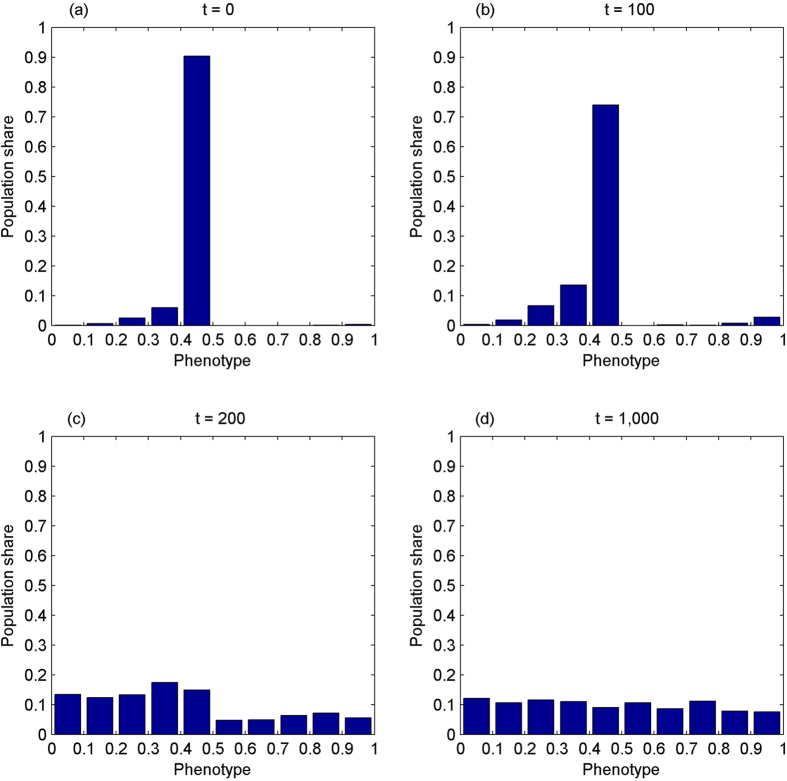
The phenotype becomes more evenly distributed without inequity aversion while the amount of cooperation opportunities is non-increasing over time. Panel (a) reproduces Panel (d) of Fig. 1. At *t* = 100, when there have been not any opportunities of cooperation *for the first time*, the perfect sense of fairness still dominates, over 70% of population falling on [0.4, 0.5], Panel (b). This is because that cooperation opportunities have been *a scarce good* for 100 generations. At *t* = 200, Panel (c), the phenotype spreads out, but is more concentrated on the left. At *t* = 1,000, Panel (d), it becomes more evenly distributed.

The second experiment simulates the evolution of the phenotype without IA while the amount of cooperation opportunities is non-increasing over time. The initial distribution of the phenotype is reproduced from Panel (d) of Fig. 1. Figure 2 shows the results, suggesting that the phenotype is degenerating gradually.

The results of the first two experiments suggest that the phenotype demonstrates a tendency of converging towards the perfect sense of fairness without IA while the prospect of cooperation is increasing over time, but degenerating towards even distribution while the prospect of cooperation is decreasing.

### An experiment without IA_2_

**Figure 3:**
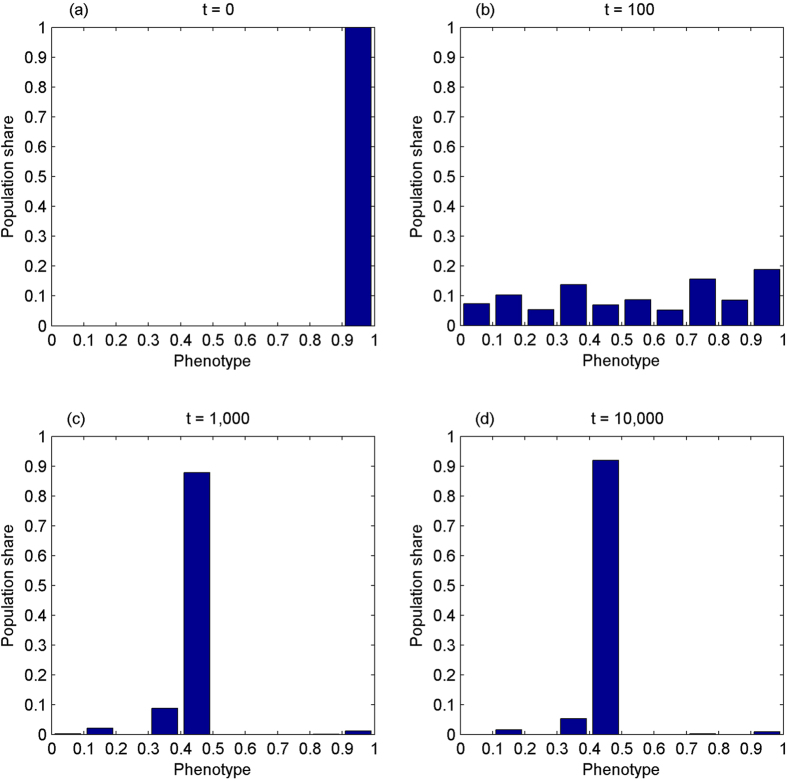
The phenotype evolves towards the perfect sense of fairness without the second degree inequity aversion while the amount of cooperation opportunities is increasing over time. It starts from the mass range of [0.9, 1], Panel (a), corresponding to nearly pure egoism. At t = 100, it has scattered unevenly across the whole range, Panel (b). At t = 1,000, Panel (c), over 80% of population concentrate on [0.4, 0.5]. It converges towards the mass range of [0.4, 0.5] at t = 10,000, accounting for over 90% of population, Panel (d).

The third experiment simulates the evolution of the phenotype in the absence of IA_2_ while the number of cooperation opportunities is increasing over time. Assume that the phenotype and γ both start from the mass range of [0.9, 1]. The latter corresponds to very low levels of IA_1_. Figure 3 shows the results.

The results show that the phenotype evolves towards the perfect sense of fairness. With endogenous IA_1_, it is interesting to see how *γ* evolves though not figured. It starts from the mass range of [0.9, 1]. At t = 100, it has scattered unevenly across the whole range. At t = 1,000, its distribution has been skewing left, over 60% of population falling on [0.7, 1] within which half population fall on [0.9, 1]. This pattern persists at t = 10,000.

Thus far, the results show that the phenotype converges towards the perfect sense of fairness without IA_2_, regardless of whether IA_1_ is present or not. Second, the left-skewness in the distribution of *γ* upon the convergence of the phenotype suggests that individuals with higher degrees of IA_1_ will gradually lose their fitness in the long term.

### An experiment with genetic IA_2_

**Figure 4:**
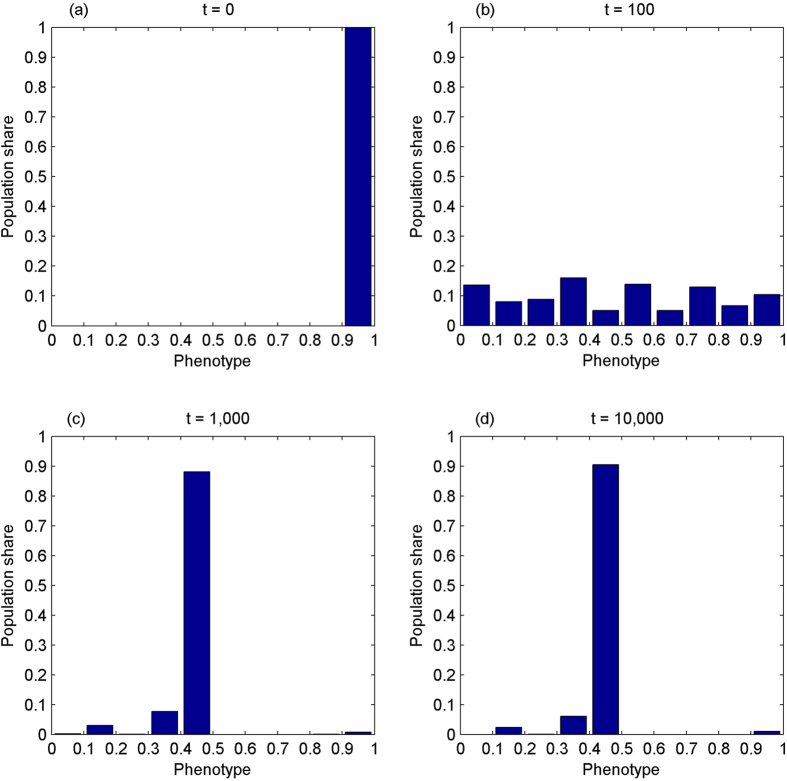
Assume that both the phenotype and IA_1_ discount factor fall on [0.9, 1], and IA_2_ falls on [0, 0.1] initially. The phenotype evolves towards the perfect sense of fairness while the amount of cooperation opportunities is increasing over time. It starts from the mass range of [0.9, 1], Panel (a). At t = 100, it has scattered across the whole range, Panel (b). At t = 1,000, Panel (c), it is highly concentrated on [0.4, 0.5], over 80% of population. It converges towards [0.4, 0.5] at t = 10,000, 90% of population, Panel (d).

The fourth experiment simulates the evolution of the phenotype with genetic IA_2_ while the number of cooperation opportunities is increasing over time. It corresponds to the case where *λ* = 0 so that the degree of IA_2_ is completely dominated by genetics. Assume that both the phenotype and *γ* fall on [0.9, 1] while *θ* falls on [0, 0.1] initially, the latter of which corresponds to low levels of IA_2_. The simulation results are shown in Fig. 4.

As Fig. 4 shows, in the context where IA_2_ is dominated by genetics, the phenotype converges towards the perfect sense of fairness when the prospect of cooperation is increasing over time.

Where γ is concerned, it evolves from the mass range of [0.9, 1] towards an even distribution. From t = 0 to t = 100, it spreads out unevenly, but slightly skewed left, with the mode falling on [0.9, 1], accounting for just over 30% of population. At t = 1,000, it becomes less skewed, nearly 80% of population are evenly distributed on [0.5, 0.6] and [0.7, 1]. At t = 10,000, it is more evenly distributed. Where *θ* is concerned, it evolves from the mass range of [0, 0.1] to right skewness. At t = 100, it has scattered unevenly across the whole range, slightly left skewed. At t = 1,000, it forms a bimodal distribution with two modes falling on [0, 0.1] and [0.5, 0.6]. At t = 10,000, it is skewed right with the mode falling on [0, 0.1], accounting for nearly 25% of the population. As well, less than 30% of the population fall on [0.6, 1].

These observations suggest that *γ* and *θ* are not mutually independent, even if a long-term valuable cooperative relationship does not exist between two partners. This implication is consistent with the hypothesis that IA_2_ requires anticipation of IA_1_ in the partner and its negative impact on the relationship^1^. Second, the distribution of *θ* is more skewed right when *γ* is more evenly distributed. To be precise, the degree of IA_2_ is converging towards [0, 0.1] when *γ* is more evenly distributed over the whole range. It supports, at least partially, the argument that sufficiently high stakes lead responder behaviour to converge towards full acceptance of low offers, even in the absence of learning^28^. Otherwise, *θ* would not converge towards [0, 0.1]. Thirdly, the results constitute a sharp contrast to the general observations from most studies on UG experiments where the majority of proposers offer 40% to 50% of the total sum, corresponding to *θ*’s range of [0.8, 1]. This contrast suggests that the human IA_2_ may be substantially influenced by social manipulation, instead of genetics alone.

### An experiment with cultural IA_2_ = 2/3

**Figure 5:**
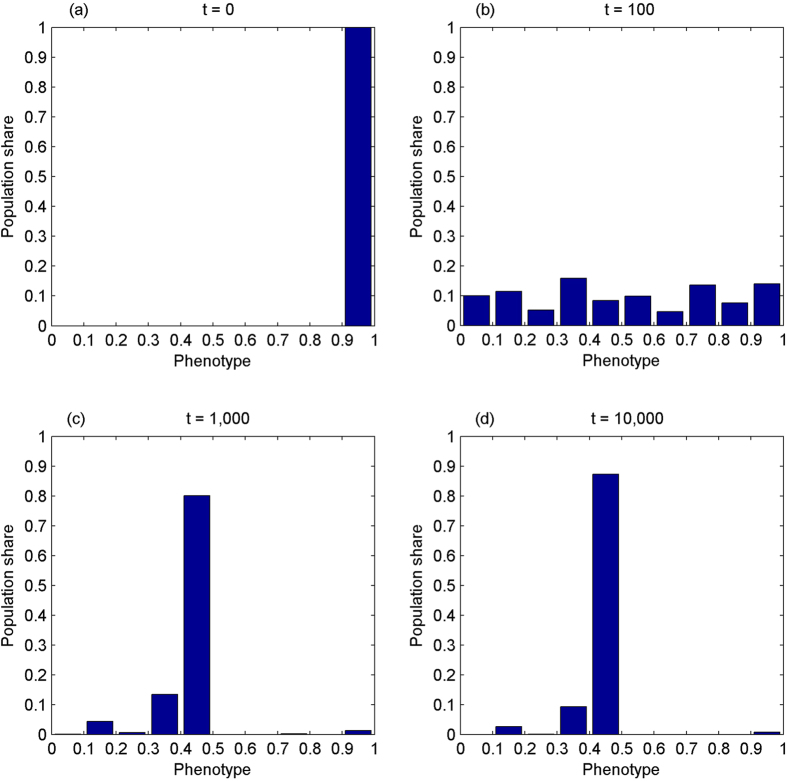
The phenotype converges towards the perfect sense of fairness when the second degree of inequity aversion is set at 2/3 while the amount of cooperation opportunities is increasing over time. The phenotype distributes evenly within [0.9, 1] at t = 0, Panel (a). It scatters unevenly across the whole range at t = 100, Panel (b). At t = 1,000, nearly 80% of population fall on [04, 0.5], Panel (c). At t = 10,000, nearly 90% of population fall on the mass range of [0.4, 0.5], Panel (d).

The fifth experiment examines the evolutionary pattern of the phenotype in the context of social manipulation dominance of IA_2_ given by *θ*_*i*_ = *θ*_*j*_ = 2/3. Under this setting, the disadvantaged partner will receive 1/3 to 1/2 of the total sum when both *b*_*i*_ + *b*_*j*_ ≤ 1 and *b*_*i*_ ≠ *b*_*j*_ hold. This setting is consonant with many experimental studies on UG^2^,^4^,^12^,^13^,^14^,^15^,^16^,^17^,^18^,^19^,^29^. Hence, the results will be highly illuminative in exploring why people redistribute voluntarily. Assume that both the phenotype and*γ* fall on [0.9, 1] initially. The simulation results are shown in Fig. 5.

With *θ* = 2/3, the phenotype converges towards the mass range of [0.4, 0.5], suggesting that people redistribute voluntarily to satisfy fairness norms, the perfect sense of fairness^35^. Where *γ* is concerned, it demonstrates the tendency of convergence towards the right end, the lowest level of IA_1_. At t = 100, it scatters unevenly across the whole range. At t = 1,000, nearly 40% (80%) of population fall on the range of [0.9, 1] ([0.7, 1]). The pattern has been persisting ever since. In contrast to the fourth experiment where *γ* is distributed more or less uniformly at *t* = 10,000, this result suggests that holding *θ* at 2/3 can effectively constrain the variation of IA_1_, thus increase the size of benefits from cooperation in the long term. The larger is the degree of social manipulation, the faster IA_1_ (*γ*) is converging towards zero (unity).

### An experiment with cultural IA_2_ = 1

**Figure 6:**
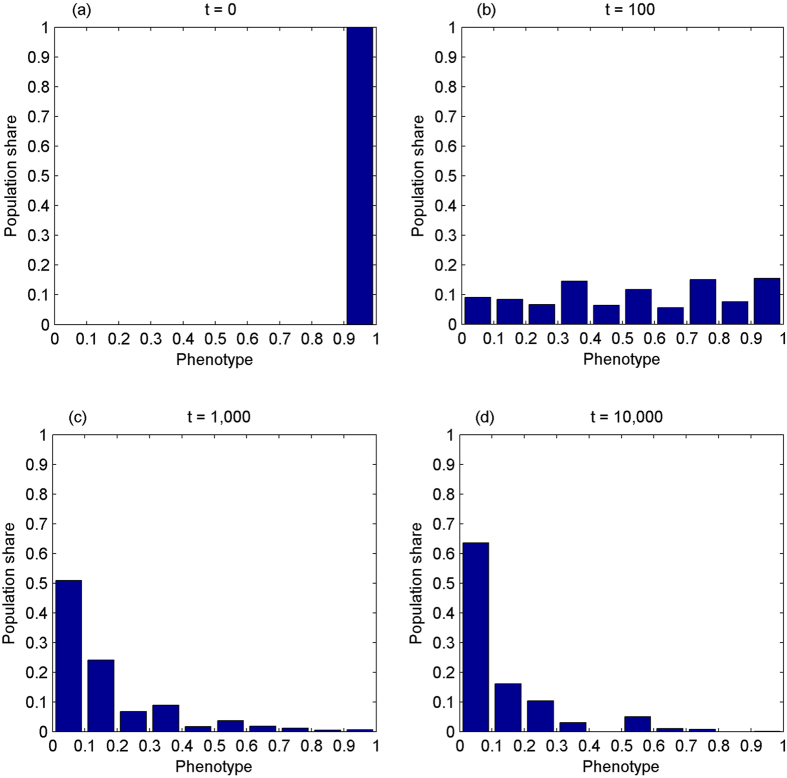
The phenotype demonstrates the tendency of convergence towards pure altruism under the perfect social manipulation dominance of the second degree inequity aversion while the amount of cooperation opportunities is increasing over time. The phenotype is evenly distributed within [0.9, 1] at t = 0, Panel (a). It scatters unevenly across the whole range at t = 100, Panel (b). At t = 1,000, over 50% (80%) of population fall on [0, 0.1] ([0, 0.3]), Panel (c). Its distribution is more skewed right at t = 10,000, over 60% (nearly 90%) of population falling on the mass range of [0, 0.1] ([0, 0.3]), Panel (d).

The final experiment examines the evolution of the phenotype in the context of the perfect social manipulation dominance of IA_2_, *θ*_*i*_ = *θ*_*j*_ = 1. Nash proposed axioms which implies that the *right* way to divide the pie is the allocation that maximizes the product of the differences from the *status quo* subject to the feasibility constraint^36^. Consider a society where Nash’s axioms serve as a guiding principle or an ethical code of conduct in the division of the total sum. The outcome of Nash bargaining equilibrium indeed corresponds to *θ*_*i*_ = *θ*_*j*_ = 1 in our setting, since the profile,(*π*_0_, *π*_0_), can be viewed as the *status quo*. Hence, the experimental simulation given *θ*_*i*_ = *θ*_*j*_ = 1 corresponds to one of the modified versions of Nash’s bargaining problem. Assume that the phenotype falls on [0.9, 1] initially. Figure 6 shows the simulation results.

As Fig. 6 shows, the phenotype converges towards pure altruism under the perfect social manipulation dominance of IA_2_. This observation is in sharp contrast to those obtained under genetic dominance of IA_2_ or cultural dominance of IA_2_ = 2/3, suggesting that there exists a threshold of cultural IA_2_, beyond which the phenotype will not converge towards the perfect sense of fairness anymore. Instead, it is altruism which would prevail.

Where *γ* is concerned, it converges towards low degree of IA_1_. At t = 100, it scatters unevenly across the whole range. At t = 1,000, over 70% (90%) of population fall on the range of [0.9, 1] ([0.7, 1]). This pattern of distribution has been persisting ever since.

Our results demonstrate that, in a society following Nash’s axioms in the division of the total sum, the phenotype will converge towards pure altruism, while IA_1_ will converge towards the low end when the prospect of cooperation is increasing over time. The tendencies of convergence towards pure altruism (or strong sense of fairness) and low degrees of IA_1_ will increase not only the probability *a prior* of a successful cooperation between two actors at each encounter, but also the expected benefit from cooperation.

### Summary and Discussion with Comments

We target a problem, the origin of human sense of fairness, and suggest a model in which phenotype will gradually converge towards the perfect sense of fairness along with the prospect of cooperation. Once the prospect of cooperation stops increasing, the sense of fairness will degenerate gradually, but it is never extinct. To some extent, the findings can justify partially the hypothesis that the human response to unfairness evolved for the sake of supporting *long-term* cooperation involving intra-generational cooperation and cooperation passing on the generation both^11^. In the environment where social manipulation of Darwinian generosity overshadows genetics, the sense of fairness could be acute to the degree of social manipulation. This is because that the higher this degree, the higher the probability *a prior* of a successful cooperation and the larger the expected benefit from cooperation will prevail, and consequently, the higher fitness will result. Furthermore, there may exist a threshold in the degree of social manipulation of Darwinian generosity, beyond which the phenotype will converge towards altruism rather than the perfect sense of fairness.

However, the evidences presented here are not convincingly supporting the above remarks if viewed from a broader perspective. Above all, the current setting is far from the reality since any topology of gene manipulation is not well mixed. Meanwhile, albeit the time course could be familiar for human societies, there is a relevant shortage of the present model. Namely, it assumes random partnership, which is just a narrow sense of cooperative relationships. As far as the gene-culture approach is concerned, in retrospect, recent development in this topic will certainly shed light on the following three new directions toward more realistic situations.

The first direction associates with the implications of the choice of discrete or continuous sets of phenotypes (or strategies). Where fairness is concerned, theoretical works mainly focus on the division strategy of UG^7^,^37^,^38^. Along with the shift in the focus to discrete or continuous sets of strategies, it shows that discrete set of strategies could lead to rich dynamical behaviours in the spatial UG^39^, followed by the finding that fine-grained strategy intervals promote the evolution of fairness^40^. To be precise, for the whole set of N^2^ coarse-grained strategies where N is a small natural number, the two strategies coexistent in the stationary state are always the two most rational strategies with the lowest possible acceptance level. As N becomes larger, the dominant strategy, by contrast, is both fair and empathetic.

Secondly, where the types of players and spatiality both are considered simultaneously, the scenario of the evolution of fairness becomes much more complicated. Within the context of either empathetic, pragmatic or independent players, in terms of the roles as the proposal or the respondent, compounded further by different topologies of either a homogeneous number of neighbors or scale-free networks, and selection rules of either natural selection or social penalty in the form of collateral punishment up to the first neighbors of the less fit players, the rejection of low offers arises in the context of natural selection regardless of the underlying topology. In the context of social punishment, however, the dynamical behaviour of the system changes radically due to the fact that the players have to consider the fitness of their neighbors in addition to their own benefit. It shows that players can adapt their offers and acceptance thresholds independently. Moreover, the dynamical equilibria of both typologies converge to the setting of pragmatic players, the framework where full altruistic behaviour is observed. Notwithstanding, it should be emphasised that the abundance of highly generous individuals observed when social penalty is at work arises from a purely scale-free effect combined with a social enforcement of altruism^41^. Experimentally, similar findings out of data collecting from nearly 200 dictators across various treatments suggest that agents do not ubiquitously choose the most selfish outcome in dictator games, implying institutions do matter substantially in reality^42^.

And lastly but most importantly, pairwise social interactions have dominated the interpretation of many biological data, though far from reality, above all in the public goods game with many players. When many players are involved, decision-making becomes more complicated for the potential increase in the number of equilibria. Group interactions can hardly be treated as the corresponding sum of pairwise interactions. As a consequence, the public goods game with many players would probably be well interpreted in terms of group interactions for their inherent irreducibility^43^. Indeed, this perspective has been applied to study the emergence of fairness in repeated group interactions, in which individuals engage in an iterated N-person prisoner’s dilemma^44^. Recently, the synergy between statistical physics, network science and evolutionary game theory has made considerable advances in the study of evolutionary dynamics of group interactions on top of structured populations, including lattices, complex networks (involving social heterogeneity, bipartite graphs, hierarchical social structure, and populations of mobile agents), and coevolutionary models^45^,^46^,^47^,^48^,^49^. As a matter of fact, it has been reviewed that the effect of spatiality could play a decisive role in general on the evolution of fairness in group interactions on structured populations^49^. The current framework can be extended accordingly to examine gene-culture coevolution wherein migration in combination with within-group selection against altruists is a much stronger force than selection between groups. Hopefully, it will probably stimulate the interest of another scientific community.

## Methods

Bargaining over a prey may involve issues of haggling, information, game structure, reputation, and trust in cooperation process^36^,^50^,^51^,^52^,^53^,^54^. Additionally, even if an agreement *a priori* were made, one would suspect how the deal goes ahead in the absence of explicit contracts and regulatory institutions. It is plausible that an altruistic individual is likely to be sanctioned by her selfish partner, and consequently resulting in detrimental effects on the cooperation itself. These negative effects have been overlooked by the prevailing self-interest approach for long. Indeed, whether trust will emerge from individuals paired by a random encounter is not only a fundamental problem in the behavioural sciences, but also reliant on the degree of fairness-based altruism.

The trust game, which assesses the amount one player invests with another in order to potentially increase total investment income with default risk, has been considered as an appropriate experimental candidate to find the properties of trust. In one class of the trust game experiments, players have revealed the preference of placing trust in members of their own age group^35^. Other studies also confirm that in-group members are evaluated more favorably than out-group members^55^. These research findings suggest that trust discrimination exists at least on the grounds of group. An experimental study which attempts to capture the relationship among trust, sanctions, and altruistic cooperation demonstrates that fairness-based altruism matters for human cooperation^3^.

In light of the above discussion, we believe that fairness-based altruism is a powerful source of human cooperation, and should be reflected in our modelling. We follow Nash’s two-person cooperative game for simplicity^36^. Assume that both players simultaneously formulate their demands before action, say *b*_*i*_, *b*_*j*_ ∈ [0, 1]. If (*b*_*i*_, *b*_*j*_) is feasible, each player at least gets what he demanded. Specifically, we assume that the constraint set is binding, such that the share player *i* gets, *β*_*i*_, is proportional to what he demanded, i.e., *β*_*i*_ = *b*_*i*_/(*b*_*i*_ + *b*_*j*_). If it is infeasible, they both will prey alone in the end for that specific encounter.

Suppose that each individual is programmed to follow a certain mode of behaviour in formulating his demand. Denote *b*_*i*,*t*_ as the share formulated by individual *i* of the *t*^th^ generation, which is considered as a phenotype serving as a measure of the human sense of fairness.

Suppress the subscript *t* temporarily for brevity. Let *d*_*i*_ ∈ [0, 1] be individual *i*’s degree of IA_1_, which can be interpreted as a measure of sanctions against revealed selfish or greedy intentions of the opponent, and is detrimental to altruistic cooperation if *d*_*i*_ > 0. Thus, fairness-based altruism is linked to human cooperation. Define *γ*_*i*_ ≡ 1 − *d*_*i*_ as the IA_1_-discount factor, and *θ*_*i*_ ∈ [0, 1] as the degree of IA_2_. Assume that (*b*_*i*_, *γ*_*i*_) is solely determined by individual *i*’s genes, (B_*i*_, Γ_*i*_) where B_*i*_ and Γ_*i*_ are all binary strings of length 4. In the case where IA is absent, B_*i*_ is extended to a binary string of length 8 to allow for more variants within population.

The condition, *b*_*i*_ + *b*_*j*_ ≤ 1, is the prerequisite that two individuals agree to hunt cooperatively. Each individual does not know the true value of *γ* of her partner before action. The IA_1_-discount factor of the disadvantaged actor, either *γ*_*i*_ or *γ*_*j*_, will be triggered during the process of cooperation, and thus causes negative reactions to inequity to the detriment of the actor. Assume that the total net reward is deflated by the IA_1_-discount factor of the disadvantaged actor.

Where *θ*_*i*_ is concerned, it matters only on occasions of inequity benefiting one individual or overcompensation. The fact that IA_2_ has only been observed in chimpanzees and humans suggests that IA_2_ requires more advanced cognition and emotional control than IA_1_ does. Social learning or culture (social manipulation) may be of substantial importance in the determination of *θ*_*i*_. Assume that *θ*_*i*_ is determined by the interplay of two inheritance systems: genetics and social manipulation^6^. The former is a bijection from Θ_*i*_, a binary string of length 4, while the latter, summarized by the social manipulation index, *α* ∈ [0, 1], reflects social norms regulating a portion of overcompensation to be transferred from the advantaged actor to the disadvantaged one. Conceptually, *α* mirrors social order^3^ or a measure of evolution of ideas, wisdom devices, or memes, the latter of which refers to behaviours and ideas copied from person to person by imitation^56^.

Assume that the value of *α* is common knowledge, but the IA_2_-related genetic inheritance index is a private information before one-shot partnering relationship ends. For simplicity, assume that the interplay between both inheritance systems has only two potential outcomes, dominated by either genetics or social manipulation. When both conditions, *b*_*i*_ + *b*_*j*_ ≤ 1 and *b*_*i*_ ≠ *b*_*j*_, meet, either *θ*_*i*_ or *θ*_*j*_ will initiate. Once initiate, the advantaged actor will make non-negative transfers perceived as fair by herself or her society to her opponent. Assume that, wherever applicable, the larger *θ*_*i*_ or *θ*_*j*_ is, the larger is the portion of overcompensation to be transferred without reducing the total net reward. The bijection from gene-culture to phenotypes is expressed as (*b*_*i*_, *γ*_*i*_, *θ*_*i*_) = (B_*i*_ × Φ, Γ_*i*_ × Φ, *λ α* + (1 − *λ*) Θ_*i*_ × Φ), where *b*_*i*_, *γ*_*i*_, *θ*_*i*_, *α* ∈ [0, 1], *λ* ∈ {0, 1}, and Φ =  (2^0^, 2^1^, 2^2^, 2^3^)^T^/(2^4^ − 1). It is clear that *λ* is a dummy variable. In case where social manipulation dominates, *λ* = 1 and *θ*_*i*_ = *α*. Otherwise, it will be determined by genetics.

Denote Π_*i*_ as individual *i*’s payoff in each random encounter with *j*. Then, Π_*i*_ will equal [*β*_*i*_ − *θ*_*i*_(*β*_*i*_ − *β*_*j*_)/2] *γ*_*j*_*π*_1_ if *b*_*i*_ + *b*_*j*_ ≤ 1 and *b*_*i*_ > *b*_*j*_ hold, or [*β*_*i*_ + *θ*_*j*_(*β*_*j*_ − *β*_*i*_)/2] *γ*_*i*_*π*_1_ if *b*_*i*_ + *b*_*j*_ ≤ 1 and *b*_*i*_ < *b*_*j*_ hold, or 0.5 *π*_1_ if *b*_*i*_ = *b*_*j*_ = 0.5 holds, or *π*_0_ if otherwise, for all *i* ≠ *j*, *i*, *j* = 1, ..., *N*. Given the setting, the number of successful cooperation during a lifetime is endogenous while subject to perturbations. Assume that the time discount factor is unity. Thus, the lifetime payoffs of individual *i* of the *t*^th^ generation, denoted as 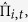 will equal 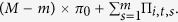


At the third stage of each generation, all individuals begin to breed. Assume that the ability to transmit one’s own genotype is proportional to his own lifetime payoffs. As a result, given *N*, the probability *a priori* of any individual *i* of the *t*^th^ generation transmitting her (his) own genotype to the next generation can be calculated, denoted as *P*_*i*,*t*+1_, i.e. 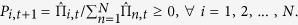


Denote (B_*i*,*t*_, Γ_*i*,*t*_, Θ_*i*,*t*_) as the *reproduced* genes of individual *i* of the *t*^th^ generation, a binary string of length 12. After all of the *N* reproduced genes have been generated across individuals, they randomly match in pairs which would give births to the *realised* genes of next generation, (B_*i*,*t*+1_, Γ_*i*,*t*+1_, Θ_*i*,*t*+1_).

Using the standard doctrine in evolutionary biology, assume that the realised genes come from two sources: genetic inheritance with high probability as well as mutation with low but positive probability. For each individual fertilized ovum, its inherited genes will have its origin from either maternal side or paternal side, or both. The former two cases occur through the genetic operator of reproduction, while the later occurs through the operator of crossover. Assume that the probability of crossover for each element of the inherited genes is *x*, *x* ∈ [0, 1]. Assume further that, for each element of the inherited genes, there is a probability of mutation, *y* ∈ [0, 1]. Therefore, each realised gene has to go through all the operators to materialize.

It is necessary to clarify at the outset that our simulations might be inconsistent with the evolutionarily stable strategy (ESS) in the short term. Stability does not necessarily coincide with the ESS or (in some cases) any of the ESS when the dynamical system is subjected to small but non-vanishing perturbations^57^. Thus, the concept of stochastic stability will be useful in considering the meaning of convergence across generations, a concept this study has heavily relied upon.

## Additional Information

**How to cite this article**: Liu, T.-G. and Lu, Y. Gene–culture interaction and the evolution of the human sense of fairness. *Sci. Rep.* **6**, 32483; doi: 10.1038/srep32483 (2016).

## Supplementary Material

Supplementary Information
