# Supplementary material for: Gene–culture interaction and the evolution of the human sense of fairness
Source: Sci Rep. 2016 Aug 26;6:32483. doi: 10.1038/srep32483 (PMC5000472; doi:10.1038/srep32483)
Supplement: Supplementary Information [file srep32483-s1.pdf]

Gene–culture interaction and the evolution of the human sense of fairness

Tru-Gin Liu[1,*] and Yao Lu[1,2]

[1]National Sun Yat-sen University, Institute of Economics, Kaohsiung, 80424, Taiwan

[2]Wuhan University, Dong Fureng School of Economic and Social Development, Beijing, 100010, China

*corresponding.trugin@mail.nsysu.edu.tw

An experiment without IA_2: Evolution of ϒ

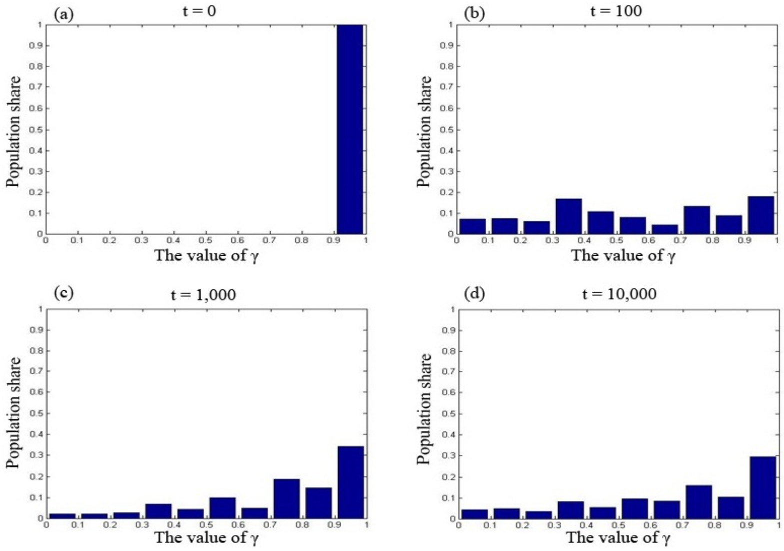

Figure 3A The distribution of the first degree inequity aversion (IA_1) discount factor, γ, evolves from the mass range of [0.9, 1] to left skewness in the absence of the second degree inequity aversion while the number of cooperation opportunities is increasing over time. In Panel (a), γ is evenly distributed within the range of [0.9, 1] at t = 0, low levels of IA_1. After 100-generation of evolution, it has scattered unevenly across the whole range, Panel (b). After another 900 generations, Panel (c), its distribution has been skewing left, over 60% of population falling on the range of [0.7, 1]. This pattern persists at 10,000 generations of evolution, Panel (d).

An experiment with genetic IA_2: Evolution of ϒ and θ

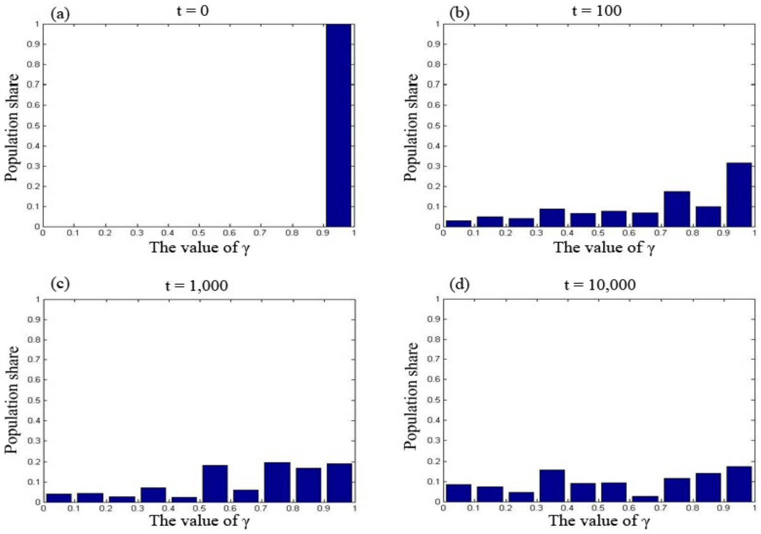

Figure 4A The distribution of the first degree inequity aversion (IA_1) discount factor, γ, evolves from the mass range of [0.9, 1] to an even distribution in the presence of the second degree inequity aversion while the number of cooperation opportunities is increasing over time. In Panel (a), γ is evenly distributed within the range of [0.9, 1] at t = 0, low levels of IA_1. It has spread out unevenly, but slightly skewed left at t = 100, with its mode falling on the range of [0.9, 1], accounting for just over 30% of population, Panel (b). At t = 1,000, the distribution becomes less skewed, nearly 80% of population are evenly distributed on the ranges of [0.5, 0.6] and [0.7, 1], Panel (c). At t = 10,000, it has become more evenly distributed, Panel (d).

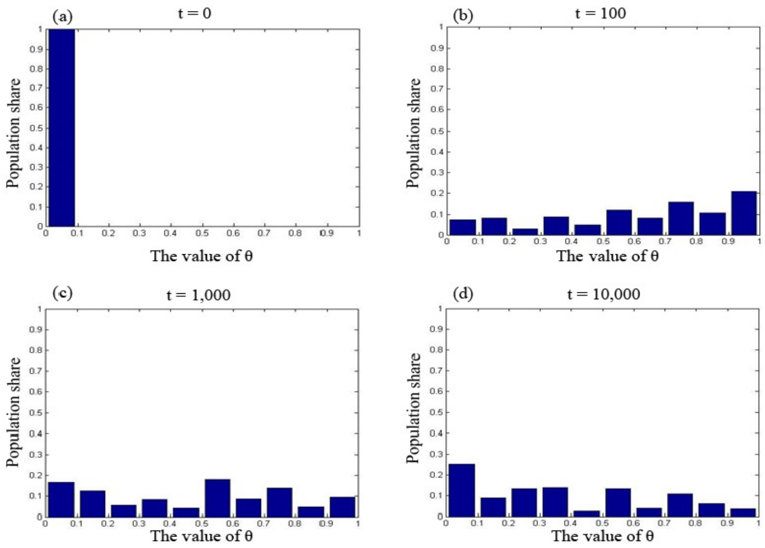

Figure 4B The distribution of the second degree inequity aversion (IA_2), θ, evolves from the mass range of [0, 0.1] to right skewness when the number of cooperation opportunities is increasing over time. In Panel (a), θ is evenly distributed within the range of [0, 0.1] at t = 0, low levels of IA_2. After 100-generation of evolution, it has scattered unevenly across the whole range, slightly left skewed indeed, see Panel (b). After another 900 generations, Panel (c), it forms a bimodal distribution with two modes falling on the ranges of [0, 0.1] and [0.5, 0.6]. After another 9,000 generations, Panel (d), the distribution of θ is skewed right with the mode falling on the range of [0, 0.1], accounting for nearly 25% of the population. Note also that less than 30% of the population fall on the range of [0.6, 1].

An experiment with cultural IA_2 = 2/3: Evolution of ϒ

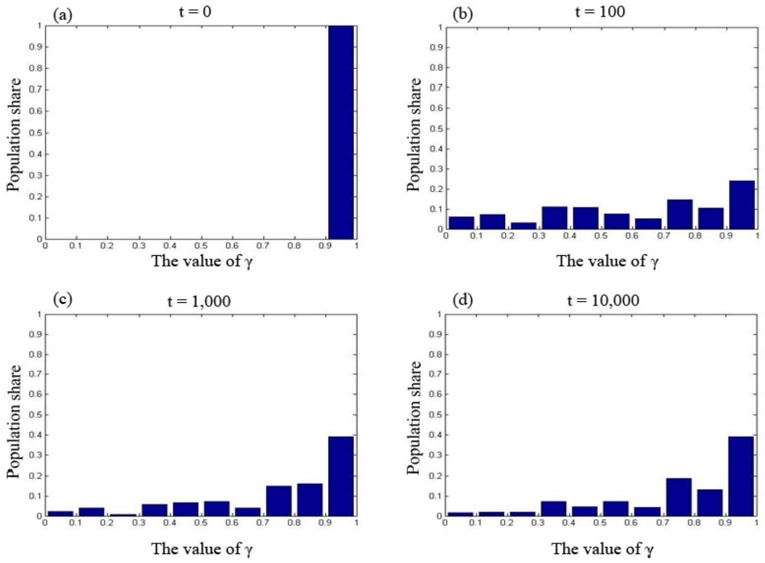

Figure 5A The distribution of the first degree inequity aversion (IA_1) discount factor, γ, demonstrates the tendency of convergence towards low degree of IA_1 when IA_2 is dominated by social manipulation at 2/3 while the number of cooperation opportunities is increasing over time. In Panel (a), γ is evenly distributed within the range of [0.9, 1] at t = 0, low levels of IA_1. At t = 100, it scatters unevenly across the whole range. At t = 1,000, nearly 40% (80%) of population fall on the range of [0.9, 1] ([0.7, 1]), Panels (c). This pattern of distribution has been persisting ever since, see Panel (d).

An experiment with cultural $IA_2 = 1$: Evolution of ϒ

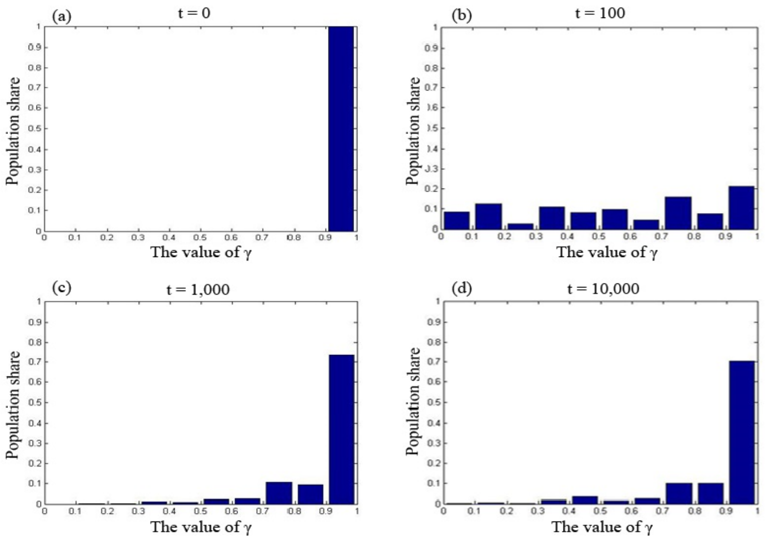

Figure 6A The distribution of the first degree inequity aversion (IA_1) discount factor, γ, demonstrates the tendency of convergence towards low degree of IA_1 under perfect social manipulation dominance of IA_2 while the number of cooperation opportunities is increasing over time. In Panel (a), γ is evenly distributed within the range of [0.9, 1] at t = 0, low levels of IA_1. At t = 100, it scatters unevenly across the whole range. At t = 1,000, over 70% (90%) of population fall on the range of [0.9, 1] ([0.7, 1]), Panels (c). This pattern of distribution has been persisting ever since, see Panel (d).

The Matlab procedure which creates data and produces Figure 1

```
%parameter assignment
f1=1;%total capture in cooperation
f0=0.01;%individual's capture when hunting singly
f3=[1;2;4;8;16;32;64;128];
f4=2^8-1;
m0=100;%total number of prey times in lifetime
m1=0;%number of attempt-cooperation times contemporary
m2=0.5;%probability of crossover
m3=0.01;%probability of mutation
m4=1000;%the number of individuals·
a=ones(m4,8);%all individuals' DNAs within population
b=a*f3/f4;%all individuals' phenotypes within population
c=a;

%auxiliary parameter assignment
f7=0;
f5=1:m4;
f6=zeros(m4,1);
q=zeros(2,m4/2);
q(1,:)=1:m4/2;
p=zeros(m4,1);
beta=zeros(m4,1);

%begining
for t=1:10000;% number of generations
  m1=floor(t/100);
  lide=zeros(m4,1);

%cooperation in each genreation
  for i1=1:m1;%the times of cooperations is m1
    q(2,:)=randsample(m4/2+1:m4,m4/2);
    for i2=1:m4/2;%cooperation is successful or failed
      if b(q(1,i2))+b(q(2,i2))<=1;%
        lide(q(1,i2))=lide(q(1,i2))+b(q(1,i2))/(b(q(1,i2))+b(q(2,i2)))*f1;
        lide(q(2,i2))=lide(q(2,i2))+b(q(2,i2))/(b(q(1,i2))+b(q(2,i2)))*f1;
      else
        lide(q(1,i2))=lide(q(1,i2))+f0;
        lide(q(2,i2))=lide(q(2,i2))+f0;
      end;
```

```
    end;%cooperation is successful or failed
  end;%the times of cooperations is m1
  lide=f0*(m0-m1)+lide;

%the auxiliary DNAs before crossover
  sumlide=sum(lide);
  p=lide/sumlide;
  f6=randsrc(m4,1,[f5;p']);
  for i5=1:m4;
    c(i5,:)=a(f6(i5),:);
  end;

%crossover
  q(2,:)=randsample(m4/2+1:m4,m4/2);
  for i6=1:m4/2;%total crossovers successful or failed
    for i7=1:8;%each crossover successful or failed
      if rand<m2;
         f7=c(q(1,i6),i7);
         c(q(1,i6),i7)=c(q(2,i6),i7);
         c(q(2,i6),i7)=f7;
      end;
    end;%each crossover successful or failed
  end;%total crossovers successful or failed

%mutation
  for i8=1:m4;
    for i9=1:8;
      if rand<m3;
         if c(i8,i9)>0;
            c(i8,i9)=0;
         else
            c(i8,i9)=1;
         end;
      end;
    end;
  end;

%DNA of next generation
  a=c;
  b=a*f3/f4;%phenotypes of next generation
end;
```

```
%draw the figure
x=[0.05 0.15 0.25 0.35 0.45 0.55 0.65 0.75 0.85 0.95];
[x1,x2]=hist(b,x);
bar(x2,x1/m4)
```

The Matlab procedure which creates data and produces Panel (b) of Figure 2

```
for t=1:100;%number of generations
  m1=m0-t;
  lide=zeros(m4,1);

%cooperation in each genreation
  for i1=1:m1;%the times of cooperations is m1
    q(2,:)=randsample(m4/2+1:m4,m4/2);
    for i2=1:m4/2;%cooperation is successful or failed
      if b(q(1,i2))+b(q(2,i2))<=1;
        lide(q(1,i2))=lide(q(1,i2))+b(q(1,i2))/(b(q(1,i2))+b(q(2,i2)))*f1;
        lide(q(2,i2))=lide(q(2,i2))+b(q(2,i2))/(b(q(1,i2))+b(q(2,i2)))*f1;
      else
        lide(q(1,i2))=lide(q(1,i2))+f0;
        lide(q(2,i2))=lide(q(2,i2))+f0;
      end;
    end;%cooperation is successful or failed
  end;%the times of cooperations is m1
  lide=f0*(m0-m1)+lide;

%the auxiliary DNAs before crossover
  sumlide=sum(lide);
    p=lide/sumlide;
  f5=1:m4;
  f6=randsrc(m4,1,[f5;p']);
  for i5=1:m4;
    c(i5,:)=a(f6(i5),:);
  end;

%crossover
  q(2,:)=randsample(m4/2+1:m4,m4/2);
  for i6=1:m4/2;%total crossovers successful or failed
    for i7=1:8;%each crossover successful or failed
      if rand<m2;
        f7=c(q(1,i6),i7);
        c(q(1,i6),i7)=c(q(2,i6),i7);
        c(q(2,i6),i7)=f7;
      end;
    end;%each crossover successful or failed
```

```
  end;%total crossovers successful or failed

%mutation
  for i8=1:m4;
    for i9=1:8;
      if rand<m3;
         if c(i8,i9)>0;
            c(i8,i9)=0;
         else
            c(i8,i9)=1;
         end;
      end;
    end;
  end;

%DNA of next generation
  a=c;
  b=a*f3/f4;%phenotypes of next generation
end;

%draw the figure
x=[0.05 0.15 0.25 0.35 0.45 0.55 0.65 0.75 0.85 0.95];
[x1,x2]=hist(b,x);
bar(x2,x1/m4)
```

****The Matlab procedure which creates data and produces Panel (c) and Panel (d) of Figure 2****

```
for t=1:100;%number of generations
  lide=zeros(m4,1);

%cooperation in each genreation
  for i1=1:m1;%the times of cooperations is m1
    q(2,:)=randsample(m4/2+1:m4,m4/2);
    for i2=1:m4/2;%cooperation is successful or failed
      if b(q(1,i2))+b(q(2,i2))<=1;
        lide(q(1,i2))=lide(q(1,i2))+b(q(1,i2))/(b(q(1,i2))+b(q(2,i2)))*f1;
        lide(q(2,i2))=lide(q(2,i2))+b(q(2,i2))/(b(q(1,i2))+b(q(2,i2)))*f1;
      else
        lide(q(1,i2))=lide(q(1,i2))+f0;
        lide(q(2,i2))=lide(q(2,i2))+f0;
      end;
    end;%cooperation is successful or failed
  end;%the times of cooperations is m1
  lide=f0*(m0-m1)+lide;

%the auxiliary DNAs before crossover
  sumlide=sum(lide);
    p=lide/sumlide;
  f5=1:m4;
  f6=randsrc(m4,1,[f5;p']);
  for i5=1:m4;
    c(i5,:)=a(f6(i5),:);
  end;

%crossover
  q(2,:)=randsample(m4/2+1:m4,m4/2);
  for i6=1:m4/2;%total crossovers successful or failed
    for i7=1:8;%each crossover successful or failed
      if rand<m2;
        f7=c(q(1,i6),i7);
        c(q(1,i6),i7)=c(q(2,i6),i7);
        c(q(2,i6),i7)=f7;
      end;
    end;%each crossover successful or failed
  end;%total crossovers successful or failed
```

```
%mutation
  for i8=1:m4;
    for i9=1:8;
      if rand<m3;
         if c(i8,i9)>0;
            c(i8,i9)=0;
         else
            c(i8,i9)=1;
         end;
      end;
    end;
  end;

%DNA of next generation
  a=c;
  b=a*f3/f4;%phenotypes of next generation
end;

%draw the figure
x=[0.05 0.15 0.25 0.35 0.45 0.55 0.65 0.75 0.85 0.95];
[x1,x2]=hist(b,x);
bar(x2,x1/m4)
```

****The Matlab procedure which creates data and produces Figure 3A and Figure 3****

```
%parameter assignment
f1=1;%total capture in cooperation
f0=0.01;%individual's capture when hunting singly
f3=[1;2;4;8]/15;
m0=100;%total number of prey times in lifetime
m1=0;%number of attempt-cooperation times contemporary
m2=0.5;%probability of crossover
m3=0.01;%probability of mutation
m4=1000;%the number of individuals
a1=ones(m4,4);
a2=a1;
b1=a1*f3;
b2=a2*f3;
c1=a1;
c2=a2;
c=[c1,c2];
aerfa=1;

%auxiliary parameter assignment
f7=0;
f5=1:m4;
f6=zeros(m4,1);
q=zeros(2,m4/2);
q(1,:)=1:m4/2;
p=zeros(m4,1);

%begining
for t=1:10000;%number of generations
  m1=floor(t/100);
  lide=zeros(m4,1);
  a=[a1,a2];

%cooperation in each genreation
  for i1=1:m1;%the times of cooperations is m1
    q(2,:)=randsample(m4/2+1:m4,m4/2);
    for i2=1:m4/2;%cooperation is successful or failed
      if b1(q(1,i2))+b1(q(2,i2))<=1;
        if b1(q(1,i2))>b1(q(2,i2));%begin to discuss  IA-1
```

```
lide(q(1,i2))=lide(q(1,i2))+b1(q(1,i2))/(b1(q(1,i2))+b1(q(2,i2)))*b2(q(2,i2))*f1;

lide(q(2,i2))=lide(q(2,i2))+b1(q(2,i2))/(b1(q(1,i2))+b1(q(2,i2)))*b2(q(2,i2))*f1;
         else
            if b1(q(1,i2))<b1(q(2,i2));

lide(q(1,i2))=lide(q(1,i2))+b1(q(1,i2))/(b1(q(1,i2))+b1(q(2,i2)))*b2(q(1,i2))*f1;

lide(q(2,i2))=lide(q(2,i2))+b1(q(2,i2))/(b1(q(1,i2))+b1(q(2,i2)))*b2(q(1,i2))*f1;
            else
               lide(q(1,i2))=lide(q(1,i2))+0.5*f1;
               lide(q(2,i2))=lide(q(2,i2))+0.5*f1;
            end;
         end;%end discussing IA-1
      else
         lide(q(1,i2))=lide(q(1,i2))+aerfa*f0;
         lide(q(2,i2))=lide(q(2,i2))+aerfa*f0;
      end;
    end;%cooperation is successful or failed
  end;%the times of cooperations is m1
  lide=f0*(m0-m1)+lide;

%the auxiliary DNAs before crossover
  sumlide=sum(lide);
  p=lide/sumlide;
  f6=randsrc(m4,1,[f5;p']);
  for i5=1:m4;
    c(i5,:)=a(f6(i5),:);
  end;

%crossover
  q(2,:)=randsample(m4/2+1:m4,m4/2);
  for i6=1:m4/2;
    for i7=1:8;
      if rand<m2;
         f7=c(q(1,i6),i7);
         c(q(1,i6),i7)=c(q(2,i6),i7);
         c(q(2,i6),i7)=f7;
      end;
    end;
```

```
  end;

%mutation
  for i8=1:m4;
    for i9=1:8;
      if rand<m3;
         if c(i8,i9)>0;
            c(i8,i9)=0;
         else
            c(i8,i9)=1;
         end;
      end;
    end;
  end;

%DNA of next generation
  a1=c(:,1:4);
  a2=c(:,5:8);
  b1=a1*f3;
  b2=a2*f3;
end;

%draw the figure
x=[0.05 0.15 0.25 0.35 0.45 0.55 0.65 0.75 0.85 0.95];
[x1,x2]=hist(b1,x);
[x3,x4]=hist(b2,x);
bar(x2,x1/m4) %draw the 3A
%bar(x4,x3/m4) %draw the 3B

%because the MATLAB can only draw a single figure each time, so the figure 3A
and 3B cannot be drawn together.
```

****The Matlab procedure which creates data and produces Figure 4A, Figure 4B and Figure 4****

```
%parameter assignment
f1=1;%total capture in cooperation
f0=0.01;%individual's capture when hunting singly
f3=[1;2;4;8]/15;
m0=100;%total number of prey times in lifetime
m1=0;% number of attempt-cooperation times contemporary
m2=0.5;%probability of crossover
m3=0.01;%probability of mutation
m4=1000;%the number of individuals
a1=ones(m4,4);
a2=a1;
a3=zeros(m4,4);
b1=a1*f3;
b2=a2*f3;
b3=a3*f3;
c1=a1;
c2=a2;
c3=a3;
c=ones(m4,12);
aerfa=1;

%auxiliary parameter assignment
f7=0;
f5=1:m4;
f6=zeros(m4,1);
q=zeros(2,m4/2);
q(1,:)=1:m4/2;
p=zeros(m4,1);

%begining
for t=1:10000;%number of generations
  m1=floor(t/100);
  lide=zeros(m4,1);
  a=c;

%cooperation in each genreation
  for i1=1:m1;%the times of cooperations is m1
```

```
   q(2,:)=randsample(m4/2+1:m4,m4/2);
   for i2=1:m4/2;%cooperation is successful or failed
     if b1(q(1,i2))+b1(q(2,i2))<=1;
        if b1(q(1,i2))>b1(q(2,i2));%begin to discuss  IA-1

lide(q(1,i2))=lide(q(1,i2))+f1*b2(q(2,i2))*(b1(q(1,i2))/(b1(q(1,i2))+b1(q(2,i2)))*(
1-b3(q(1,i2))/2)+b1(q(2,i2))/(b1(q(1,i2))+b1(q(2,i2)))*b3(q(1,i2))/2);

lide(q(2,i2))=lide(q(2,i2))+f1*b2(q(2,i2))*(b1(q(2,i2))/(b1(q(1,i2))+b1(q(2,i2)))*(
1-b3(q(1,i2))/2)+b1(q(1,i2))/(b1(q(1,i2))+b1(q(2,i2)))*b3(q(1,i2))/2);
        else
          if b1(q(1,i2))<b1(q(2,i2));

lide(q(1,i2))=lide(q(1,i2))+f1*b2(q(1,i2))*(b1(q(1,i2))/(b1(q(1,i2))+b1(q(2,i2)))*(
1-b3(q(2,i2))/2)+b1(q(2,i2))/(b1(q(1,i2))+b1(q(2,i2)))*b3(q(2,i2))/2);

lide(q(2,i2))=lide(q(2,i2))+f1*b2(q(1,i2))*(b1(q(2,i2))/(b1(q(1,i2))+b1(q(2,i2)))*(
1-b3(q(2,i2))/2)+b1(q(1,i2))/(b1(q(1,i2))+b1(q(2,i2)))*b3(q(2,i2))/2);
          else
            lide(q(1,i2))=lide(q(1,i2))+0.5*f1;
            lide(q(2,i2))=lide(q(2,i2))+0.5*f1;
          end;
        end;%end discussing IA-1
     else
        lide(q(1,i2))=lide(q(1,i2))+aerfa*f0;
        lide(q(2,i2))=lide(q(2,i2))+aerfa*f0;
     end;
   end;%cooperation is successful or failed
  end;%the times of cooperations is m1
  lide=f0*(m0-m1)+lide;

%the auxiliary DNAs before crossover
  sumlide=sum(lide);
  p=lide/sumlide;
  f6=randsrc(m4,1,[f5;p']);
  for i5=1:m4;
    c(i5,:)=a(f6(i5),:);
  end;

%crossover
  q(2,:)=randsample(m4/2+1:m4,length(q));
```

```
 for i6=1:m4/2;
  for i7=1:12;
   if rand<m2;
      f7=c(q(1,i6),i7);
      c(q(1,i6),i7)=c(q(2,i6),i7);
      c(q(2,i6),i7)=f7;
   end;
  end;
 end;

%mutation
 for i8=1:m4;
  for i9=1:12;
   if rand<m3;
      if c(i8,i9)>0;
         c(i8,i9)=0;
      else
         c(i8,i9)=1;
      end;
   end;
  end;
 end;

%DNA of next generation
 a1=c(:,1:4);
 a2=c(:,5:8);
 a3=c(:,9:12);
 b1=a1*f3;
 b2=a2*f3;
 b3=a3*f3;
end;
%draw the figure
x=[0.05 0.15 0.25 0.35 0.45 0.55 0.65 0.75 0.85 0.95];
[x1,x2]=hist(b1,x);
[x3,x4]=hist(b2,x);
[x5,x6]=hist(b3,x);
bar(x2,x1/m4) %draw the 4C
%bar(x4,x3/m4) %draw the 4A
%bar(x6,x5/m4) %draw the 4B
%because the MATLAB can only draw a single figure each time, so the figure 4A
and 4B and 4C cannot be drawn together.
```

The Matlab procedure which creates data and produces Figure 5A and Figure 5

```
%parameter assignment
f1=1;%total capture in cooperation
f0=0.01;%individual's capture when hunting singly
f3=[1;2;4;8]/15;
m0=100;%total number of prey times in lifetime
m1=0;% number of attempt-cooperation times contemporary
m2=0.5;%probability of crossover
m3=0.01;%probability of mutation
m4=1000;%the number of individuals
a1=ones(m4,4);
a2=a1;
a3=a1;
b1=a1*f3;
b2=a2*f3;
b3=a3*f3*2/3;
c1=a1;
c2=a2;
c3=a3;
c=ones(m4,12);
aerfa=1;

%auxiliary parameter assignment
f7=0;
f5=1:m4;
f6=zeros(m4,1);
q=zeros(2,m4/2);
q(1,:)=1:m4/2;
p=zeros(m4,1);

%begining
for t=1:10000;%number of generations
  m1=floor(t/100);
  lide=zeros(m4,1);
  a=c;

%cooperation in each genreation
  for i1=1:m1;%the times of cooperations is m1
```

```
    q(2,:)=randsample(m4/2+1:m4,m4/2);
    for i2=1:m4/2;%cooperation is successful or failed
      if b1(q(1,i2))+b1(q(2,i2))<=1;
        if b1(q(1,i2))>b1(q(2,i2));%begin to discuss IA-1

lide(q(1,i2))=lide(q(1,i2))+f1*b2(q(2,i2))*(b1(q(1,i2))/(b1(q(1,i2))+b1(q(2,i2)))*(
1-b3(q(1,i2))/2)+b1(q(2,i2))/(b1(q(1,i2))+b1(q(2,i2)))*b3(q(1,i2))/2);

lide(q(2,i2))=lide(q(2,i2))+f1*b2(q(2,i2))*(b1(q(2,i2))/(b1(q(1,i2))+b1(q(2,i2)))*(
1-b3(q(1,i2))/2)+b1(q(1,i2))/(b1(q(1,i2))+b1(q(2,i2)))*b3(q(1,i2))/2);
        else
          if b1(q(1,i2))<b1(q(2,i2));

lide(q(1,i2))=lide(q(1,i2))+f1*b2(q(1,i2))*(b1(q(1,i2))/(b1(q(1,i2))+b1(q(2,i2)))*(
1-b3(q(2,i2))/2)+b1(q(2,i2))/(b1(q(1,i2))+b1(q(2,i2)))*b3(q(2,i2))/2);

lide(q(2,i2))=lide(q(2,i2))+f1*b2(q(1,i2))*(b1(q(2,i2))/(b1(q(1,i2))+b1(q(2,i2)))*(
1-b3(q(2,i2))/2)+b1(q(1,i2))/(b1(q(1,i2))+b1(q(2,i2)))*b3(q(2,i2))/2);
          else
            lide(q(1,i2))=lide(q(1,i2))+0.5*f1;
            lide(q(2,i2))=lide(q(2,i2))+0.5*f1;
          end;
        end;%end discussing IA-1
      else
        lide(q(1,i2))=lide(q(1,i2))+aerfa*f0;
        lide(q(2,i2))=lide(q(2,i2))+aerfa*f0;
      end;
    end;%cooperation is successful or failed
  end;%the times of cooperations is m1
  lide=f0*(m0-m1)+lide;

%the auxiliary DNAs before crossover
  sumlide=sum(lide);
  p=lide/sumlide;
  f6=randsrc(m4,1,[f5;p']);
  for i5=1:m4;
    c(i5,:)=a(f6(i5),:);
  end;

%crossover
  q(2,:)=randsample(m4/2+1:m4,length(q));
```

```
  for i6=1:m4/2;
    for i7=1:8;
      if rand<m2;
        f7=c(q(1,i6),i7);
        c(q(1,i6),i7)=c(q(2,i6),i7);
        c(q(2,i6),i7)=f7;
      end;
    end;
  end;

%mutation
  for i8=1:m4;
    for i9=1:8;
      if rand<m3;
        if c(i8,i9)>0;
          c(i8,i9)=0;
        else
          c(i8,i9)=1;
        end;
      end;
    end;
  end;

%DNA of next generation
  a1=c(:,1:4);
  a2=c(:,5:8);
  a3=c(:,9:12);
  b1=a1*f3;
  b2=a2*f3;
end;

%draw the figure
x=[0.05 0.15 0.25 0.35 0.45 0.55 0.65 0.75 0.85 0.95];
[x1,x2]=hist(b1,x);
[x3,x4]=hist(b2,x);
[x5,x6]=hist(b3,x);
bar(x2,x1/m4) %draw the 5A
%bar(x4,x3/m4) %draw the 5
%because the MATLAB can only draw a single figure each time, so the figure 5A
and 5 cannot be drawn together.
```

The Matlab procedure which creates data and produces Figure 6A and Figure 6

```
%parameter assignment
f1=1;%total capture in cooperation
f0=0.01;%individual's capture when hunting singly
f3=[1;2;4;8]/15;
m0=100;%total number of prey times in lifetime
m1=0;% number of attempt-cooperation times contemporary
m2=0.5;%probability of crossover
m3=0.01;%probability of mutation
m4=1000;%the number of individuals
a1=ones(m4,4);
a2=a1;
a3=a1;
b1=a1*f3;
b2=a2*f3;
b3=a3*f3;
c1=a1;
c2=a2;
c3=a3;
c=ones(m4,12);
aerfa=1;

%auxiliary parameter assignment
f7=0;
f5=1:m4;
f6=zeros(m4,1);
q=zeros(2,m4/2);
q(1,:)=1:m4/2;
p=zeros(m4,1);

%begining
for t=1:10000;%number of generations
  m1=floor(t/100);
  lide=zeros(m4,1);
  a=c;

%cooperation in each genreation
  for i1=1:m1;%the times of cooperations is m1
    q(2,:)=randsample(m4/2+1:m4,m4/2);
```

```
   for i2=1:m4/2;%cooperation is successful or failed
     if b1(q(1,i2))+b1(q(2,i2))<=1;
       if b1(q(1,i2))>b1(q(2,i2));%begin to discuss IA-1

lide(q(1,i2))=lide(q(1,i2))+f1*b2(q(2,i2))*(b1(q(1,i2))/(b1(q(1,i2))+b1(q(2,i2)))*(
1-b3(q(1,i2))/2)+b1(q(2,i2))/(b1(q(1,i2))+b1(q(2,i2)))*b3(q(1,i2))/2);

lide(q(2,i2))=lide(q(2,i2))+f1*b2(q(2,i2))*(b1(q(2,i2))/(b1(q(1,i2))+b1(q(2,i2)))*(
1-b3(q(1,i2))/2)+b1(q(1,i2))/(b1(q(1,i2))+b1(q(2,i2)))*b3(q(1,i2))/2);
       else
         if b1(q(1,i2))<b1(q(2,i2));

lide(q(1,i2))=lide(q(1,i2))+f1*b2(q(1,i2))*(b1(q(1,i2))/(b1(q(1,i2))+b1(q(2,i2)))*(
1-b3(q(2,i2))/2)+b1(q(2,i2))/(b1(q(1,i2))+b1(q(2,i2)))*b3(q(2,i2))/2);

lide(q(2,i2))=lide(q(2,i2))+f1*b2(q(1,i2))*(b1(q(2,i2))/(b1(q(1,i2))+b1(q(2,i2)))*(
1-b3(q(2,i2))/2)+b1(q(1,i2))/(b1(q(1,i2))+b1(q(2,i2)))*b3(q(2,i2))/2);
         else
           lide(q(1,i2))=lide(q(1,i2))+0.5*f1;
           lide(q(2,i2))=lide(q(2,i2))+0.5*f1;
         end;
       end;%end discussing IA-1
     else
       lide(q(1,i2))=lide(q(1,i2))+aerfa*f0;
       lide(q(2,i2))=lide(q(2,i2))+aerfa*f0;
     end;
   end;%cooperation is successful or failed
  end;%the times of cooperations is m1
  lide=f0*(m0-m1)+lide;

%the auxiliary DNAs before crossover
  sumlide=sum(lide);
  p=lide/sumlide;
  f6=randsrc(m4,1,[f5;p']);
  for i5=1:m4;
    c(i5,:)=a(f6(i5),:);
  end;

%crossover
  q(2,:)=randsample(m4/2+1:m4,length(q));
  for i6=1:m4/2;
```

```
    for i7=1:8;
      if rand<m2;
         f7=c(q(1,i6),i7);
         c(q(1,i6),i7)=c(q(2,i6),i7);
         c(q(2,i6),i7)=f7;
      end;
    end;
  end;

%mutation
  for i8=1:m4;
    for i9=1:8;
      if rand<m3;
         if c(i8,i9)>0;
            c(i8,i9)=0;
         else
            c(i8,i9)=1;
         end;
      end;
    end;
  end;

%DNA of next generation
  a1=c(:,1:4);
  a2=c(:,5:8);
  a3=c(:,9:12);
  b1=a1*f3;
  b2=a2*f3;
  b3=a3*f3;
end;

%draw the figure
x=[0.05 0.15 0.25 0.35 0.45 0.55 0.65 0.75 0.85 0.95];
[x1,x2]=hist(b1,x);
[x3,x4]=hist(b2,x);
[x5,x6]=hist(b3,x);
bar(x2,x1/m4) %draw the 6A
%bar(x4,x3/m4) %draw the 6

%because the MATLAB can only draw a single figure each time, so the figure 6A
and 6 cannot be drawn together.
```
